# Neonatal Lupus Erythematosus as a Rare Cause of Fever in Young Infants

**DOI:** 10.3390/jcm10143195

**Published:** 2021-07-20

**Authors:** Ji Yeon Song, Su Eun Park, Joung-Hee Byun, Narae Lee, Young Mi Han, Shin Yun Byun, Seong Heon Kim

**Affiliations:** 1 Department of Pediatrics, Pusan National University Children’s Hospital, Yangsan 50612, Korea; bestsongped@gmail.com (J.Y.S.); psepse@naver.com (S.E.P.); africa3217@naver.com (J.-H.B.); whitecloud11@hanmail.net (N.L.); skybluehym@gmail.com (Y.M.H.); byun410@hanmail.net (S.Y.B.); 2 Department of Pediatrics, Seoul National University Children’s Hospital & College of Medicine, Seoul 03080, Korea

**Keywords:** neonatal lupus erythematosus, fever, infant, anti-SSA/Ro, anti-SSB/La, SLE, Sjögren’s syndrome

## Abstract

Neonatal lupus erythematosus (NLE) is a rare disease caused by passively transmitted autoantibodies from the mother. NLE is a multi-organ system disease characterized by cutaneous, cardiac, hematological, hepatobiliary, and neurological manifestations. This study aimed to review the various symptoms and clinical manifestations in young infants with NLE and their mothers. We conducted a retrospective review of medical records of patients with NLE who were both examined and treated at Pusan National University Children’s Hospital between January 2009 and December 2020 and their mothers. Twenty-seven patients with NLE comprising 13 male patients (48.1%) and 14 female patients (51.9%) were included. The most common symptom was rash (40.7%), followed by fever (25.9%), arrhythmia (14.8%), splenomegaly (11.1%), and intrauterine growth retardation (7.4%). Seven patients with fever had various organ system manifestations, including cutaneous (100%), hematological (71.4%), hepatobiliary (57.1%), and central nervous system (CNS; 28.6%) manifestations. Two of the febrile patients had aseptic meningitis. Cutaneous, cardiac, hematological, hepatobiliary, and CNS involvement were noted in 44.4%, 18.5%, 51.9%, 40.7%, and 22.2% of the patients, respectively. Systemic lupus erythematosus (SLE) was the most common maternal disease (14/27, 51.9%). Ten mothers (37.0%) had not been diagnosed with any autoimmune disease until their babies were diagnosed. Among them, three were subsequently diagnosed with SLE, five were diagnosed with the Sjögren’s syndrome, and two of them still had no known diagnosis of any autoimmune disorder. Fever is a common symptom of NLE; thus, when there is no clear focus of fever in infants, NLE needs to be considered, especially in cases with skin rashes.

## 1. Introduction

Neonatal lupus erythematosus (NLE) is a rare and acquired autoimmune disease caused by passive placental transfer of maternal autoantibodies, especially anti-SSA/Ro and anti-SSB/La antibodies [1,2]. The most common presenting symptom of NLE is rash, and clinical manifestations of NLE include cutaneous, cardiac, hepatobiliary, hematologic, and central nervous system (CNS) manifestations [3,4]. While fever is a common symptom of various autoimmune diseases, autoimmune diseases are not usually considered as a differential diagnosis in newborns or young infants, unlike in older children. There have been few reports of fever being the presenting symptom in NLE [5]; however, we have encountered several febrile infants whose fever had no known etiology, and who were subsequently diagnosed with NLE. To summarize the various clinical presentations of NLE, this study reviewed the presenting clinical features of infants with NLE and of their mothers from 2009 to 2020.

## 2. Materials and Methods

We conducted a retrospective cohort study of all patients with NLE who presented to the Pusan National University Children’s Hospital between January 2009 and December 2020. The diagnosis of NLE was based on the presence of serum anti-SSA/Ro or anti-SSB/La in an infant born to a mother with a rheumatic disease or in an infant with clinical features (rash, cardiac, hepatic, hematologic manifestations, CNS, or other manifestations). Demographic data, clinical manifestations, laboratory findings, and history of maternal rheumatologic disease were collected from the medical records of the young infants and their mothers. Laboratory investigations included tests for antibodies such as anti-Ro/SSA and anti-La/SSB; complete blood count examination for white blood cell, hemoglobin, and platelet levels; and liver function tests (LFTs) including tests for alanine aminotransferase (ALT), aspartate aminotransferase (AST), alkaline phosphatase, and bilirubin. Echocardiography and electrocardiography (ECG) were performed to confirm the cardiac manifestations. Anemia, thrombocytopenia, and neutropenia were considered at or below the 2.5th percentile, respectively, by age and sex, based on the recommendation of the American Academy of Pediatrics. The normal ranges of LFTs were defined by the 21st edition of the Nelson Textbook of Pediatrics. Elevated AST/ALT levels were deemed to be instances of hepatobiliary manifestation. Aseptic meningitis was characterized by pleocytosis and increased protein levels and by negative in Gram staining, routine culture, and polymerase chain reaction (PCR) of the cerebrospinal fluid (CSF). We collected and summarized the data and performed a descriptive analysis in this study. This study was approved by the Institutional Review Board (IRB) of Pusan National University Yangsan Hospital (approval No. 05-2021-076), and the need for consent was waived.

## 3. Results

### 3.1. Demographic Information and Presenting Symptoms of NLE

Twenty-seven patients with NLE were included in this study. The patient characteristics are presented in Table 1. There were 13 males (48.1%) and 14 females (51.9%) among the 27 cases. The average gestational age (GA) at delivery was 38.0 ± 3.0 weeks (median 39.0 weeks; range 28 + 1–41 + 5 weeks), with 14.8% preterm births. The mean birth weight was 2823 ± 724.0 g (median 3070 g; range 720–3660 g) and small for GA, comprising 22.2% of the cases. The onset of clinical symptoms ranged from birth to 128 days, and the mean age at diagnosis of NLE was 28 days. The most common symptom was rash (40.7%), followed by fever (25.9%), arrhythmia (14.8%), splenomegaly (11.1%), and intrauterine growth retardation (IUGR; 7.4%). Seven patients (25.9%) were asymptomatic.

### 3.2. Patients with NLE Presenting with Fever

All seven patients who had fever had cutaneous manifestations. Hematologic, hepatobiliary, and CNS involvement were detected in five (71.4%), four (57.1%), and two (28.6%) febrile patients, respectively. (Table 2). Cardiac involvement was not observed. The average fever duration was 1.7 ± 0.95 days (median: 1 day; range: 1–3 days). Of the seven patients with fever, six had a rash at the time of diagnosis. One patient (patient no. 6 in Table 2) was referred to our hospital for fever with a history of recurrent aseptic meningitis. During an evaluation for immune deficiency, we found low complement levels and decided to check for Ro/La antibodies; the anti-Ro was positive. All young infants with fever underwent blood culture, urine culture, and viral PCR studies; all tests were negative. Rare conditions were subsequently considered, and we tested for several autoantibodies, which confirmed the clinical impression. Five of the seven patients with fever underwent a lumbar puncture, and CSF analysis was performed; two of these five patients were subsequently diagnosed with aseptic meningitis.

### 3.3. Organ Involvement in Patients with NLE

Table 3 presents the various patterns of organ system involvement in patients with NLE. In this study, the most common manifestations were hematological manifestations (51.9%), and the most frequent feature was anemia (18.5%), followed by thrombocytopenia (11.1%) and neutropenia (11.1%). Combinations of hematologic dyscrasias were also observed, with some patients having anemia with neutropenia (7.4%) and thrombocytopenia with neutropenia (3.7%). In all patients, the hematologic changes were asymptomatic, transient, and spontaneously resolved the laboratory findings without morbidity. Cutaneous lesions were observed in 12 patients (44.4%). The most common finding was annular erythema, and all NLE-related rashes were self-limiting without sequelae. For examination for cardiac involvement, 24 of the 27 patients with NLE (88.9%) had ECGs, 13 patients (48.1%) had echocardiograms, and 3 patients (11.1%) underwent 24-h Holter monitoring. Cardiac manifestations included congenital heart block (11.1%) and cardiac structural abnormalities (7.4%) such as an atrial septal defect (ASD) with a ventricular septal defect (VSD) (3.7%) and a patent ductus arteriosus (PDA; 3.7%). A patient with a third-degree AV block required a pacemaker, which was placed at 4 months of age and was maintained subsequently. The patient with ASD and VSD had regular follow-up. Another patient with PDA was born premature (gestational age: 28 + 1 weeks) with an extremely low birth weight (720 g) and died at 8 days of age from respiratory distress syndrome with pulmonary hypertension and germinal matrix hemorrhage (GMH) with intraventricular hemorrhage (IVH). Hepatobiliary manifestations, elevated liver enzymes, and transaminase elevation with splenomegaly were identified in 11 (40.7%), 8 (29.6%), and 3 (11.1%) of the 27 patients, respectively. All patients with liver involvement were transient and fully resolved within 3 months after birth. CNS manifestations were identified in six patients (22.2%). Two patients (7.4%) were diagnosed with aseptic meningitis while evaluating the cause of fever. Four patients (14.8%) underwent brain ultrasonography to rule out thrombocytopenia-induced intracranial hemorrhage. Three patients (11.1%) were found to have bilateral GMH grade I with cystic changes and one patient (3.7%) had GMH grade II with IVH. None of the patients with GMH grade I with cystic changes had sequelae but one patient with GMH grade II with IVH died due to complications of prematurity.

### 3.4. Antibody Prevalence in Patients with NLE

We noted that 20 (74.1%), 15 (55.6%), 2 (7.4%), and 10 (37.0%) patients were positive for antinuclear antibody, only anti-Ro/SSA, only anti-La/SSB, and both anti-Ro/SSA and anti-La/SSB, respectively.

### 3.5. Maternal Diagnosis in Patients with NLE

Fourteen mothers (51.9%) had prior diagnosis of systemic lupus erythematosus (SLE). Two (7.4%) were diagnosed with the Sjögren’s syndrome, one (3.3%) was diagnosed with mixed connective tissue disease, and ten (37.0%) were asymptomatic. After their infants were diagnosed with NLE, three (11.1%) of the ten initially undiagnosed mothers subsequently developed SLE, five (18.5%) of them were diagnosed with Sjögren’s syndrome, and two (7.4%) of them still had no known diagnosis of any autoimmune disorder (Table 4).

## 4. Discussion

NLE is a rare neonatal disease representing the effect of transplacental passage of maternal autoantibodies, such as anti-Ro/SSA or anti-La/SSB [1,2]. The incidence of NLE is approximately 1 in 12,500 to 20,000 live births, and NLE has been reported to occur in approximately 2% of the children born to mothers with anti-Ro and anti-La antibodies [5,6]. The incidence of SLE has been increasing annually during the last decade in Korea; several studies worldwide have also reported that the incidence of SLE has increased significantly over several decades [7,8,9]. In particular, the incidence of SLE in Korea was found to be the highest in the 30–39-year age group, which is of childbearing age; therefore, the incidence of NLE may also increase.

Many studies have reported that the symptoms of NLE mainly include rash, arrhythmia, and hepatitis [3,4]. In other reports, there were higher rates of IUGR, low birth weight, or prematurity in infants of mothers with SLE or Sjögren’s syndrome [10,11]. Although fever is a common symptom of many autoimmune diseases, association with fever in NLE is not well known. There are only few case reports on fever presenting in NLE [12,13]. In our study, however, fever was the second most common presenting symptom (25.9%), and all patients with fever presented with cutaneous lesions (Table 4). Although the mechanism of fever in NLE remains unknown, an association between the presence of anti-Ro (SSA)/La (SSB) antibodies and cutaneous vasculitis has been reported [14,15]. Consequently, fever in patients with NLE may be caused by cutaneous vasculitis or other inflammatory reactions. Further studies are necessary to clarify the relevance of fever in NLE. Therefore, NLE may also be considered as a differential diagnosis in infants without a focus on fever.

Organ involvement in NLE consists of cutaneous, cardiac, hematological, hepatic, and CNS abnormalities. Cutaneous and cardiac manifestations are the most common presentations of NLE [3,4]. In this study, the most common manifestations were hematological (51.9%) and cutaneous involvement (44.4%). The most frequent feature of hematology was anemia (18.5%), followed by thrombocytopenia (11.1%) and neutropenia (11.1%). Anti-Ro antibodies bind directly to neutrophil and cause neutropenia, which is different from lymphopenia commonly found in adult patients with SLE [16]. CNS involvement in patients with NLE is rare and variable [17]. Most reported CNS manifestations were diagnosed as asymptomatic and transient hydrocephalus and macrocephaly, based on computed tomography and brain ultrasonography [18,19,20]. Some studies have reported ischemic stroke and vasculitis with discernible symptoms and severe sequelae [21,22,23], but there have been few case reports of aseptic meningitis in NLE [5]. In this study, neurologic diseases were present in six patients (22.2%), where two patients (22.2%) presented with aseptic meningitis and three patients (11.1%) had bilateral GMH grade I with cystic change, all of which had no symptoms and sequalae, but one patient (3.7%) had bilateral GMH grade II with IVH, who died due to complications of prematurity, including respiratory distress syndrome with pulmonary hypertension and PDA. Five of the seven patients with fever underwent a lumbar puncture, and subsequent CSF analysis revealed that two of these had aseptic meningitis. All patients with aseptic meningitis presented with fever and cutaneous lesions, which resolved without sequelae. These findings suggest that patients with NLE with persistent fever can be considered for CSF analysis to confirm CNS involvement, such as aseptic meningitis.

Mothers of patients with NLE present with variable manifestations. In this study, 51.9%, 7.4%, and 3.7% of the mothers of infants with NLE had SLE, Sjögren’s syndrome, and mixed connective tissue disease before pregnancy, respectively. According to some reports, about 25–58% of the mothers of NLE patients were asymptomatic [1,24,25,26]. In our study, ten (37%) of the NLE mothers had not been diagnosed with any diseases before their infants were diagnosed with NLE, three (11.1%) of them were diagnosed with SLE and five (18.5%) of them had Sjögren’s syndrome after confirmation of their infants’ NLE, and two (7.4%) of them had not yet been diagnosed. Some studies have reported that mothers who have infants with NLE have a high risk of cardiac or cutaneous manifestation in future pregnancies [16,23], and the incidence of heart or skin lesions is higher in mothers with high titers of anti-Ro/SSA or anti-La/SSB than in those with low titers of these antibodies [27,28,29]. If the mother, whose child has been diagnosed with NLE, has tested negative for autoantibodies such as Ro/La, other rare conditions should also be considered [30].

This study had several limitations. This was a retrospective study, and there were limitations in obtaining all the necessary data on the symptoms or possible manifestations of the patients. Not all patients underwent neuroimaging, ECG, or echocardiography. Furthermore, due to the retrospective nature of the study, information in the medical records may be incomplete and data be subject to bias. Therefore, more prospective studies are needed to further describe the clinical manifestations of NLE. Additionally, NLE is a rare disease, and this study included a small number of patients from a single center. Therefore, large multicenter studies are needed to identify differences by race and ethnicity. Finally, most patients with NLE were followed up for a short duration in our study. Because most patients in our study were followed up for only a short period of time, our study risks being unable to observe future sequelae in our patients with NLE.

## 5. Conclusions

Young infants with NLE present with variable symptoms such as fever, rash, arrhythmia, and splenomegaly. While there have been many reports of NLE being associated with cutaneous, cardiac, hepatobiliary, and hematologic manifestations, there are few reports on fever as a presenting symptom. In this study, 25.7% of the patients with NLE presented with fever, and two of them were diagnosed with aseptic meningitis. The result of this study suggests that we need to suspect NLE in young infants with non-infectious fever and rash where no other focus for fever could be ascertained. In cases where NLE is diagnosed, unnecessary evaluations and treatment, such as antibiotic use, may be avoided.

## Figures and Tables

**Table 1:** Demographics and presenting symptoms of NLE (*n* = 27).

		No.	Percent (%)
Sex	Male	13	48.1
	Female	14	51.9
Gestational age	≥37 weeks	23	85.2
	<37 weeks	4	14.8
Birth weight	AGA	21	77.8
	SGA	6	22.2
Onset of presenting symptoms	At birth	10	37.0
	0–4 weeks	5	18.5
	>4 weeks	5	18.5
Presenting symptoms	Fever	7	25.9
	Rash	11	40.7
	Arrhythmia	4	14.8
	Splenomegaly	3	11.1
	IUGR	2	7.4
	No symptoms	7	25.9

AGA: appropriate for gestational age; SGA: small for gestational age; IUGR: intrauterine growth retardation.

**Table 2:** NLE patients with fever (*n* = 7).

No.	Sex.	Age (Days)	Gestational Age (Weeks)	Birth Weight (Grams)	Presenting Symptom	Fever Period (Days)	Organ Involvement	Anti-Ro/La	Maternal Disease
1	M	3	38 + 0	3460	Fever, rash	1	Cutaneous, Hematologic, Hepatobiliary	+/+	SLE (new)
2	M	3	41 + 5	2860	Fever, rash	1	Cutaneous, Hematologic, Hepatobiliary	+/+	Sjögren’s syndrome (new)
3	M	1	39 + 0	3070	Fever, rash, arrhythmia	1	Cutaneous, Hematology, Hepatobiliary	+/+	SLE
4	F	5	37 + 2	2150	Fever, rash	3	Cutaneous, Hematologic, Hepatobiliary	+/−	SLE
5	M	66	37 + 0	3480	Fever, rash	3	Cutaneous, CNS	+/+	Sjögren’s syndrome (new)
6	M	63	39 + 0	3660	Fever	2	Cutaneous, Hematologic, CNS	+/−	Sjögren’s syndrome (new)
7	F	128	39 + 1	3100	Fever, rash	1	Cutaneous	+/−	Unknown

CNS: central nervous system; SLE: systemic lupus erythematosus.

**Table 3:** Organ involvement of patients with NLE (*n* = 27).

Organ		No.	Percent (%)
Cutaneous (rash)	Total	12	44.4
Cardiac involvement	Total	5	18.5
Congenital heart block	1st degree AV block	1	3.7
	2nd degree AV block	1	3.7
	3rd degree AV block	1	3.7
Cardiac structural abnormality	ASD with VSD	1	3.7
	PDA	1	3.7
Hematological involvement	Total	14	51.9
	Anemia	5	18.5
	Thrombocytopenia	3	11.1
	Neutropenia	3	11.1
	Anemia with neutropenia	2	7.4
	Thrombocytopenia with neutropenia	1	3.7
Hepatobiliary involvement	Total	11	40.7
	Transaminase elevation	8	29.6
	Transaminase elevation with splenomegaly	3	11.1
CNS involvement	Total	6	22.2
	Meningitis	2	7.4
	GMH	4	14.8

AV: atrioventricular; ASD: atrial septal defect; VSD: ventricular septal defect; PDA: patent ductus arteriosus; GMH: germinal matrix hemorrhage.

**Table 4:** Maternal diagnosis of NLE patients (*n* = 27).

	No.	Percent (%)
Undiagnosed	10	37.0
Newly diagnosed SLE	3	11.1
Newly diagnosed Sjögren’s syndrome	5	18.5
Unknown maternal disease	2	7.4
Known SLE	14	51.9
Known Sjögren’s syndrome	2	7.4
Known mixed connective tissue disease	1	3.7

SLE: systemic lupus erythematosus.
